# Exploring the Mechanism of Action of *Trachelospermi Caulis* et Folium for Depression Based on Experiments: Combining Network Pharmacology and Molecular Docking

**DOI:** 10.1155/2022/3945063

**Published:** 2022-11-30

**Authors:** Lingmiao Wen, Yuqing Fan, Wei Xiong, Yanjun Liu, Tinglan Zhang, Guihua Wei, Alvin Altamirano, Tian-e Zhang, Zhiyong Yan

**Affiliations:** ^1^School of Life Science and Engineering, Southwest Jiaotong University, Chengdu, China; ^2^Institute of Chinese Materia Medica, China Academy of Chinese Medical Sciences, Beijing, China; ^3^Department of Chemistry and Biochemistry, Northern Arizona University, Flagstaff, AZ, USA; ^4^School of Basic Medicine, Chengdu University of Traditional Chinese Medicine, Chengdu, China

## Abstract

**Objective:**

To reveal the safety, efficacy, and mechanism of action of *Trachelospermi Caulis* et Folium (TCEF) for treating depression.

**Methods:**

The maximum dose method was employed to evaluate the safety of TCEF, and its antidepressant activity was assessed using the tail suspension and sugar water depletion tests. The main components of TCEF were determined using ultrahigh performance liquid chromatography coupled with quadrupole exactive orbitrap mass spectrometer (UHPLC-Q-EOMS). The active ingredients and their action targets were obtained using network pharmacology with SwissADME and SwissTargetPrediction screening, and the targets of depression were obtained using GeneCards, DrugBank, etc. The drug and depression-related targets were intersected and analyzed *via* PPI network, GO, and KEGG. Subsequently, the binding ability of the core components of TCEF to the core targets was validated *via* molecular docking and simulation.

**Results:**

No statistically significant difference was observed between the normal and TCEF groups in terms of body weight, visceral index, and biochemical parameters (*P* > 0.05). Compared with the model group, all dose groups of TCEF had reduced the immobility time of tail suspension (*P* < 0.05) and increased the rate of sugar water consumption (*P* < 0.05). UHPLC-Q-EOMS was employed to identify 59 major components of TCEF, and network pharmacology analysis was used to screen 48 active components of TCEF for treating depression, corresponding to 139 relevant targets, including ALB, AKT1, TNF, ESR1, and CTNNB1. The involved pathways include neuroactive ligand-receptor interaction. The molecular docking results indicated that the core components have a good binding activity to the core targets.

**Conclusions:**

TCEF is a relatively safe antidepressant medicine that exerts therapeutic effects through multiple components, targets, and pathways, providing a new idea and theoretical basis for future use of TCEF to treat depression.

## 1. Introduction

Depression is a mood disorder in which the patient is in a state of loss for a long period. The disease is characterized by chronic, recurrent episodes, mainly manifesting as emotional dysfunction, excessive negative emotions, delayed thinking, irritability, loss of appetite, abnormal behavior, cognitive dysfunction, and, in severe cases, suicidal tendencies [1, 2]. These symptoms jeopardize people's lives and work and affect their physical and mental health. At present, more than 300 million people worldwide suffer from depression. With social development, the prevalence of depression has been on the rise in recent years due to the high social pressure on people [3, 4]. However, the pathogenesis of depression has not been conclusively established and is generally believed to be related to various neurochemicals as well as neuroendocrine, immune, and psychosocial factors [5, 6]. The clinical medications for depression include tricyclic antidepressants, monoamine oxidase inhibitors, selective 5-hydroxytryptamine or norepinephrine reuptake inhibitors, and dual 5-hydroxytryptamine and norepinephrine reuptake inhibitors [7, 8]. Although these drugs exert good therapeutic effects, most of them have adverse effects, such as cardiotoxicity and sleep disorders, which severely reduce patients' quality of life. Therefore, novel drugs are needed to meet these clinical demands [9].

Traditional Chinese medicine, with its safety and effectiveness, fewer side effects, and holistic treatment, has highlighted its unique advantages in preventing and treating depression, which includes rattan herbs [10–14]. *Trachelospermi Caulis* et Folium (TCEF) is a dried leafy vine stem of *Trachelospermum jasminoides* (Lindl.) Lem. in the family Oleaceae. At present, studies have demonstrated that TCEF can dispel wind and promote blood circulation, cool blood, and subdue swelling; moreover, it has anti-inflammatory and analgesic, antifatigue. Although it is also a type of rattan, relatively few studies have been conducted on its treatment of depression, and comprehensive studies on its antidepressant mechanism of action are lacking [15]. As some rattan herbs have certain toxicity, it is necessary to examine the safety of TCEF before evaluating its antidepressant effect [16–18]. Acute toxicity experimental study is a method to evaluate the safety of drugs. In this study, the acute toxicity experiment of TCEF was carried out by the maximum dosing method, that is, a single dose at the maximum allowed dose under the premise of reasonable maximum dosing concentration and dosing volume, to initially evaluate the safety of TCEF administration. Next, to study the antidepressant efficacy of TCEF, a model of depression was established using the chronic unpredictable mild stress (CUMS), and the effect of TCEF in the treatment of depression was evaluated by the sugar-water depletion test and the tail suspension test, two commonly used methods to detect the degree of depression. It is noteworthy that Chinese herbs generally exert their therapeutic effects through multiple components. The ultrahigh performance liquid chromatography coupled with quadrupole exactive orbitrap mass spectrometer (UHPLC-Q-EOMS) has the advantage of rapid and simple identification of complex components of Chinese herbs and can be used for the qualitative study of natural products in Chinese herbs compound formulas, therefore, the identification data of UHPLC-Q-EOMS were used as the basis for the subsequent network pharmacological analysis in this study [19]. In addition, traditional pharmacological studies are difficult to systematically and completely elucidate the mechanism of action of traditional Chinese herbs through multitarget and multipathway treatment of diseases, so network pharmacology is rapidly developing. Network pharmacology is based on the theory of systems biology, which fully elucidates the laws and mechanisms of interaction between pharmacodynamic substances and an organism from the perspective of systems biology and biological network equilibrium [20, 21]. Molecular docking technology is a process based on structural molecular biology to find the optimal conformation of small-molecule ligands at the active site of target proteins and to predict their binding modes, which can initially simulate the molecular mechanism of action [22, 23].

**Figure 1:**
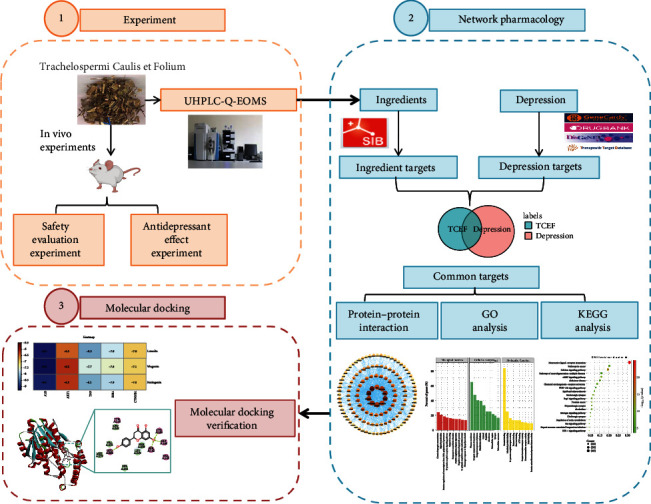
Detailed flowchart of the study design. (1) Experiments. In vivo experiments were conducted to explore the safety and efficacy of *Trachelospermi Caulis* et Folium (TCEF) in the treatment of depression. Ultrahigh performance liquid chromatography coupled with quadrupole exactive orbitrap mass spectrometer (UHPLC-Q-EOMS) analyzed the chemical ingredients contained in TCEF. (2) Network pharmacology explores the mechanism of action of TCEF in the treatment of depression. Based on the ingredients obtained by UHPLC-Q-EOMS, the active ingredients and targets of TCEF were obtained by Swiss database, and the disease targets of depression were obtained by using GeneCards, OMIM, etc. The interaction network was constructed for pharmacodynamic and disease targets to explore the key targets of TCEF for depression. The Kyoto Encyclopedia of Genes and Genomes (KEGG) and Gene Ontology (Go) were used to analyze biological processes and related pathways. (3) Molecular docking. Molecular docking to verify the binding ability of core components and targets.

Therefore, in this study, we first evaluated the safety of TCEF and its efficacy in treating depression through animal experiments. Then, we used UHPLC-Q-EOMS to characterize the chemical composition of TCEF. Next, we combined network pharmacology and molecular docking techniques to conduct a preliminary study on the mechanism of multicomponent, multitarget, and multipathway treatment of depression in TCEF. The research workflow is shown in Figure 1.

## 2. Materials and Methods

### 2.1. Animals

SPF-grade adult Kunming mice (62 males and 20 females, weighing 18–20 g) were provided by Chengdu Dashuo Experimental Animal, and the production license is SCXK (Sichuan) 2020-030. The breeding temperature was 22 °C ± 2 °C, the relative humidity was 50% ± 10%, and the light-dark cycle was 12 h. The animals were provided with food and water *ad libitum* and acclimatized for 1 week. Animal care and study protocols were approved by the institutional animal ethics committee at Southwest Jiaotong University (Approval No. SWJTU-1810-001). Ketamine (100 mg/kg) and xylazine (5 mg/kg) were administered intraperitoneally for anesthesia. Mice were euthanized with a lethal dose of pentobarbital sodium (100 mg/kg).

### 2.2. Reagents and Instruments

TCEF (Anhui Guanghe Chinese Medicine Co., Ltd., Lot No. 4439-1710024). Fluoxetine Hydrochloride Dispersible Tablets (Eli Lilly Suzhou Pharmaceutical Co., Ltd., Lot No. 7057948). A suspended tail meter (Chengdu Taimeng Software Co., Ltd.). Automatic Biochemistry Analyzer HF-220 (Manufacturer: Jinan Han Fang Medical Devices Co., Ltd.). AST (No. 20190313), ALT (No. 20190318), TP (No. 20190703), BUN (No. 20190320), UA (No. 20190323), and CRE Reagents (No. 20190328), purchased from Shandong Collison Biological Co.

### 2.3. Preparation of Drugs

The decoction was boiled thrice for 60, 30, and 30 min, respectively, and the filtrate was combined and concentrated to 50 mL *via* evaporation and dried at 105°C for 24 h to obtain the infusion. Before the experiment, configured to the required dose, the concentrations required were 0.74 g/mL for the safety evaluation experiment and 0.52 g/mL (high-dose group), 0.26 g/mL (medium-dose group), and 0.13 g/mL (low-dose group) for the depression experiment. The positive drug was fluoxetine, ground fluoxetine tablets, and added the appropriate amount of distilled water to 0.00026 g/mL concentration.

### 2.4. TCEF Safety Evaluation Experiment

A total of 40 SPF-grade Kunming mice were randomly divided into two groups, the normal group, and the TCEF group, with 10 males and females per group. The mice were acclimatized for 1 week. The normal group was gavaged with distilled water according to the maximum dose administered (0.4 mL/10 g), and the TCEF group was gavaged with TCEF at the maximum dosing concentration (0.74 g/mL). During the 14 days of continuous observation after dosing, the mice were weighed on days 1, 7, and 14. After 14 days, blood was removed, and the blood was left to stand for 30 min and then centrifuged for 15 min at 4°C and 4 000 r/min using a tabletop high-speed frozen centrifuge. The upper serum was collected, and the indicators of the liver (ALT, AST, and TP) and renal functions (CRE, UA, and BUN) were measured. After the animals were sacrificed, their hearts, livers, spleens, lungs, and kidneys were collected. The visceral index was calculated.

### 2.5. TCEF Antidepressant Effect Experiment

#### 2.5.1. Model Establishment and TCEF Treatment

A total of 42 SPF-grade Kunming male mice were randomly divided into six groups, with each group having seven mice. The normal group was not treated at all. The model, positive, TCEF low-dose, TCEF medium-dose, and TCEF high-dose groups were used to establish a depression model using the chronic unpredictable mild stress (CUMS) combined with the solitary feeding method. The stimulation methods included food and water fasting, moist bedding, restraint, forced swimming, tail pinning, and shaking of the rat cage [24]. Except for the normal group, one stimulus was randomly applied daily during the modeling period, and each stimulus was not repeated on 2 consecutive days for 4 weeks. After successful modeling, gavage was started. The low-dose, medium-dose, and high-dose groups were administered 1.3, 2.6, and 5.2 g/kg of TCEF, respectively. The positive group was administered fluoxetine at 0.0026 g/kg. The normal and model groups were administered equal amounts of pure water in a volume of 10 mL/kg for 14 days, once daily.

#### 2.5.2. Behavioral Testing

The tail suspension and sugar water depletion tests were conducted 4 weeks after modeling and 2 weeks after the drug administration to evaluate the effect of depression mice model preparation and the antidepressant effect of TCEF. For the tail suspension test, the mice were hung upside down on a special mouse tail suspension apparatus. The data were recorded after 2 min of adapting to the environment. The background of the apparatus was black, the experimenter walked as quietly as possible to avoid noise interference, and the immobility of mice within 4 min was recorded [25]. For the sugar water consumption test, two bottles of 1% sucrose solution were administered to the mice on day 1 of the experiment. On day 2, one bottle of 1% sucrose solution and one bottle of pure water were simultaneously administered, and the position of the water bottle was switched every 3–5 h. On day 3, the mice fasted with water and food for 24 h. On day 4, one bottle of 1% sucrose solution and one bottle of pure water were administered, and the consumption of 1% sucrose solution and pure water was recorded after 1 h; in addition, the sugar water preference was calculated using the following equation:

Sugar water preference index = 1%sucrose solution consumption/(1%sucrose solution consumption + pure water consumption) × 100% [26].

### 2.6. UHPLC-Q-EOMS of TCEF

The chemical composition in TCEF was obtained *via* UHPLC-Q-EOMS. Ultimate 3000 Ultra Performance Liquid Chromatography System (Dionex Corporation, USA). Thermo Gold C_18_ Chromatographic columns (100 mm × 2.1 mm, 1.9 *μ*m, Thermo Fisher Scientific, USA). The chromatographic conditions were as follows: column temperature was 40°C; flow rate, 0.5 mL/min; injection volume, 4 *μ*L; and mobile phase, 0.1% formic acid aqueous solution (a)–acetonitrile (b). A gradient elution sequence was used: 0–0.01 min, 5% B; 0.01–15 min, 5–30% B; 15–35 min, 30–95% B; 35–37 min, 95% B; 37–37.1 min, 95–5% B.

The mass spectrometry conditions were as follows: the UHPLC system was equipped with a Q-Exactive Orbitrap MS (Thermo Fisher Scientific, USA). The ion source was HESI. Positive and negative ion detection modes were used, and the spray voltage was 3500/−3000 V. The capillary temperature was 320°C; sheath gas, 35 L/min; auxiliary gas, 10 L/min. Can mode: full MS/dd-MS2, full MS resolution 70 000, dd-MS2 resolution 17 500; scan range m/z 100–1 500; and collision energy used was 40 eV in the MS/MS mode. The compounds were characterized using the Xcalibur 4.0 software and Compound Discoverer 3.0 software (Thermo Fisher Scientific, Inc., USA).

### 2.7. Network Pharmacology Analysis

#### 2.7.1. Active Ingredient and Target Screening of TCEF

The chemical components obtained using the UHPLC-Q-EOMS method were identified using the PubChem (https://pubchem.ncbi.nlm.nih.gov/) database to obtain their SDF structures and were uploaded to the SwissADME (http://www.swissadme.ch/) database for screening based on the high GI value of gastrointestinal absorption and meeting two or more “YES” of the five indices of drug similarity [27]. The screened compounds were uploaded to the small-molecule target prediction database SwissTargetPrediction (http://www.swisstargetprediction.ch/), with the species limited to human origin (*Homo sapiens*). After removing the duplicate proteins, the standard names of the proteins were obtained by the correction of the UniProt database (https://www.uniprot.org/).

#### 2.7.2. TCEF-Active Ingredient-Target Network

To investigate the interrelationship between chemical components and action targets in TCEF, candidate compounds, and their potential targets were imported into the Cytoscape 3.8.2 software to construct a network. The “degree” indicates the number of nodes connected to the nodes in the network, and those with higher-degree values may become potential targets of the compounds. The “network analyzer” function was used to analyze the Betweenness Centrality, Closeness Centrality, and Degree.

#### 2.7.3. Disease Target Screening

The keyword “Depression” was used in GeneCards (https://www.genecards.org/), DrugBank (https://go.drugbank.com/), DisGeNet (https://www.disgenet.org/), and Therapeutic Target Database (TTD, http://db.idrblab.net/ttd/) for disease gene targets. The retrieved disease targets were combined and deduplicated.

#### 2.7.4. PPI Network

Venn analysis (Venny 2.1.0) was conducted to obtain drug–disease common targets. The common targets were imported into the String (https://string-db.org) database, with the species limited to *Homo sapiens*, to obtain a protein–protein interaction (PPI) network map of the common targets of TCEF and depression. Then, the Cytoscape 3.8.2 software was imported for network analysis to obtain the key targets of TCEF for depression treatment.

#### 2.7.5. GO and KEGG Functional Enrichment Analyses

The Database of Annotated Biological Information (DAVID, https://david.ncifcrf.gov) is a large-scale gene or protein enrichment tool that can identify the most significantly enriched biological annotations. The common targets were imported into the DAVID and background database, and the gene list was restricted to “*Homo sapiens*” and then subjected to gene ontology (GO) functional enrichment and Kyoto Encyclopedia of Genes and Genomes (KEGG) pathway enrichment analyses.

### 2.8. Molecular Docking

The SDF structure of the compound was downloaded from the PubChem database, saved in the mol 2 format, and then saved in the pdbqt format after assigning charges and setting rotatable keys using AutoDockTools 1.5.6. The 3D crystal structure of the target protein was downloaded from PDB (https://www.rcsb.org), PyMol was used to remove the water molecules of the target protein, the original ligand and receptor were separated, and the target protein was imported into AutoDockTools 1.5.6 for hydrogenation and charge assignment and saved in the pdbqt format file. Molecular docking tests were conducted using AutoDockVina 1.1.2 on the core compounds and the top five targets in the degree ranking in TCEF. Docking results were visualized using Discovery Studio 3.5 Client.

### 2.9. Statistical Analysis

All data are shown as mean ± standard deviation (x ± sd). Significant differences between groups were analyzed using one-way analysis of variance (ANOVA). Statistical analysis and plots were performed using SPSS 22.0 and Graphpad Prism 8.0.2 software, respectively. *P* values <0.05 were considered statistically significant.

## 3. Results

### 3.1. Safety Evaluation Experiment

**Figure 2:**
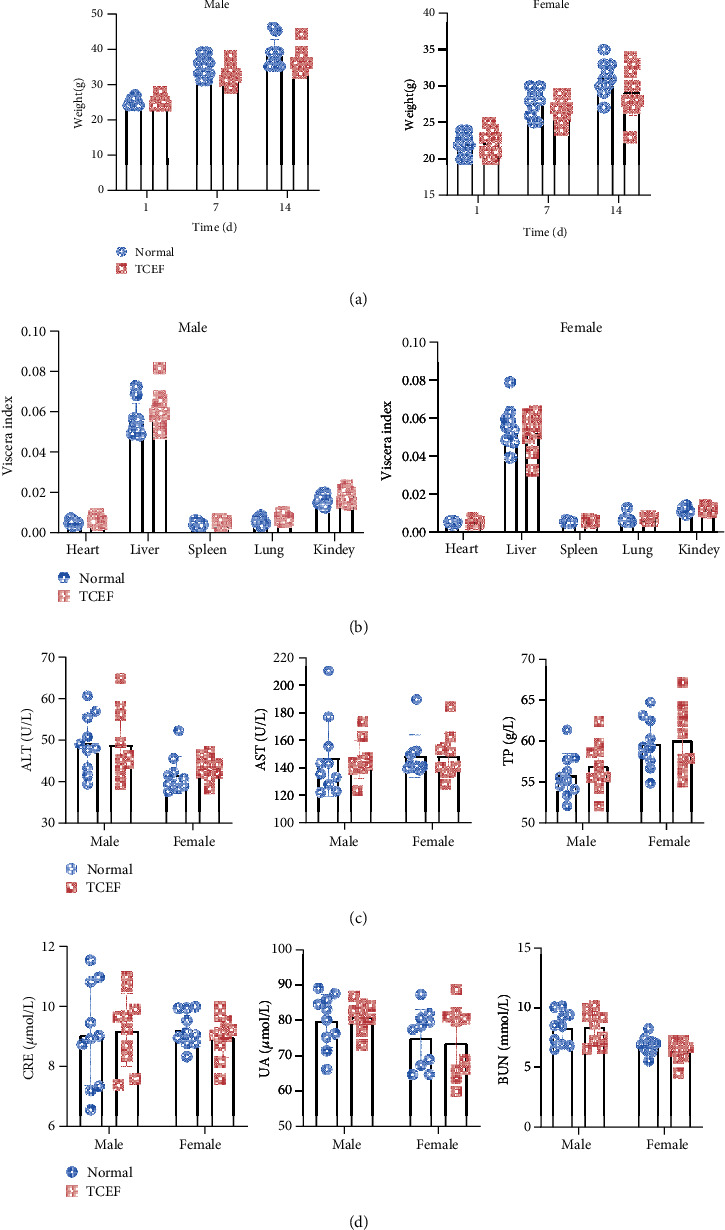
TCEF safety evaluation experiment. (a) Effect of TCEF on the body weight of mice. (b) Effect of TCEF on the viscera index of mice. (c) Effect of TCEF on the liver functions of mice (ALT, AST, and TP). (d) Effect of TCEF on the renal functions of mice (CRE, UA, and BUN) (*n* = 20 per group).

There were no obvious abnormalities or death during the 14-day observation period. In addition, no significant abnormalities were observed in food intake, mental status, and behavioral activities, and there was no significant acceleration or weakening of respiratory status, no hair loss, and no particular significant changes in appearance. As presented in Figure 2(a), there was no significant difference in body weight between the normal and TCEF groups on days 1, 7, and 14 (*P* > 0.05). The effects of TCEF on the heart, liver, spleen, lung, and kidney of mice were insignificantly different (Figure 2(b)). Moreover, there was no significant difference in ALT, AST, TP, CRE, UA, and BUN between the normal and TCEF groups (Figures 2(c) and 2(d)).

### 3.2. Antidepressant Evaluation Experiment

**Figure 3:**
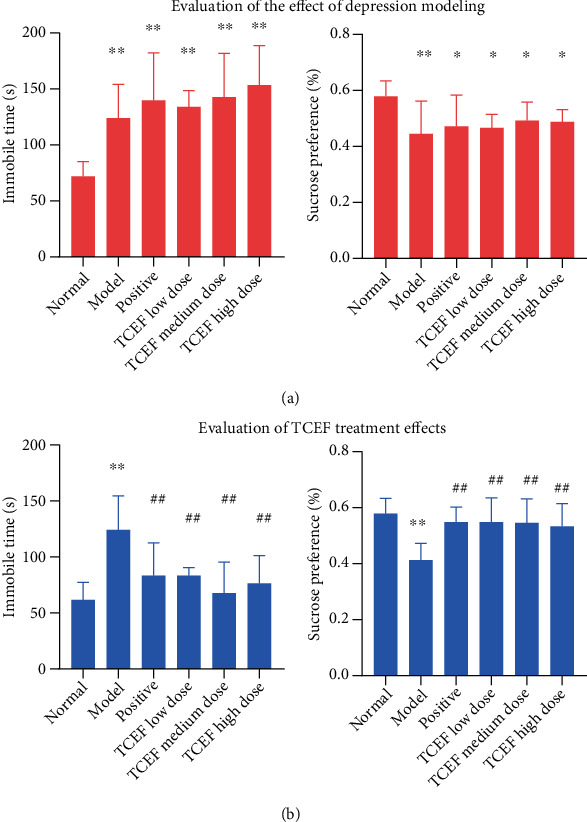
TCEF safety antidepressant effect evaluation experiment. (a) Tail suspension immobility time and sugar water preference index of mice after modeling. (b) Effect of TCEF on the immobility time of tail suspension and sugar water preference index in mice. Values are expressed as mean ± SD (*n* = 7). Compared with the normal group, ^∗^*P* < 0.05, ^∗∗^*P* < 0.01; compared with the model group, ^#^*P* < 0.05, ^##^*P* < 0.01.

As presented in Figure 3(a), in the tail suspension immobility test, compared with the normal group, the mice in each modeling group, including the positive, TCEF low-dose, medium-dose, and high-dose groups, had higher tail suspension immobility time within 4 min (*P* < 0.05) and lower sugar water consumption rate (*P* < 0.05), indicating that this experiment successfully replicated the depression model after 4 weeks of solitary feeding and modeling stimulation and could continue the experimental study of TCEF antidepressant. After 14 days of continuous administration, compared with the normal group, the mice in the model group showed an increase in tail suspension immobility time within 4 min (*P* < 0.01) and a decrease in sugar water consumption rate (*P* < 0.01); moreover, compared with the model group, the mice in the positive, TCEF low-dose, medium-dose, and high-dose groups had decreased tail suspension immobility time within 4 min (*P* < 0.01), and an increase in the sugar water consumption rate (*P* < 0.01) indicated that TCEF has antidepressant effects (Figure 3(b)).

### 3.3. Identification of the Chemical Composition of TCEF

**Figure 4:**
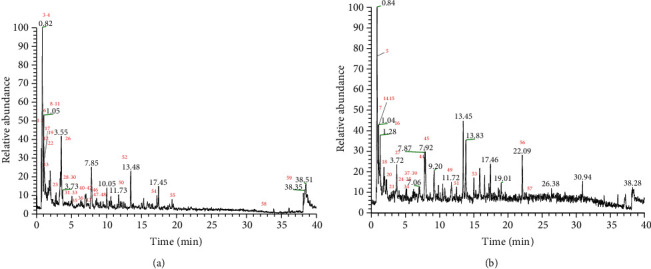
TIC diagram of TCEF. (a) TIC diagram of TCFE in positive ion mode. (b) TIC diagram of TCFE in negative ion mode.

UHPLC-Q-EOMS was employed to identify and analyze the total ion chromatograms (TIC) of TCEF in positive and negative ion scan modes, respectively, as presented in Figure 4. A total of 59 compounds were identified from TCEF according to molecular weight, retention time (RT), major Fragment Ions, and mass error by mzCloud and mzVault online databases, as listed in Table 1.

### 3.4. Chemical Constituents and Targets Related to TCEF

**Figure 5:**
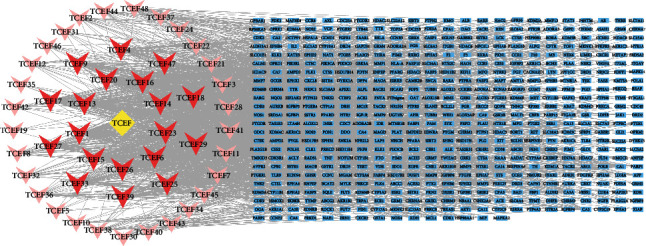
TCEF chemical composition target network. The yellow diamond indicates TCEF; the triangle, the chemical composition of TCEF (the deeper the color, the greater the degree value); and the blue rectangle, the target.

We conducted an in-depth evaluation of the properties of 59 compounds from TCEF related to absorption, distribution, metabolism, and excretion using the online tool SwissADME. SwissADME predictions indicated that 53 compounds met Lipinski's rule of five and GI absorption for high pharmacological properties [28] (Supplementary Table 1). The SwissTargetPrediction database was searched with the condition probability >0, and 48 compounds were found to be eligible, corresponding to 544 targets (Supplementary Table 2). The Cytoscape 3.8.2 software created a network diagram of the active ingredient target of action associated with TCEF (Figure 5). In this network, there were 593 nodes and 1686 edges, with each edge representing the interaction between the potential active component and the target, and the average degree value was 5.7. The degree value of a node represents the number of edges connected to that node. According to the filtering principle of network topology, a larger degree value can reflect the highly involved connected components. The top three components are luteolin (TCEF20), wogonin (TCEF1), and naringenin (TCEF13). This suggests that the three chemical components of TCEF play a more important role in depression treatment.

### 3.5. TCEF Targets for the Treatment of Depression

**Figure 6:**
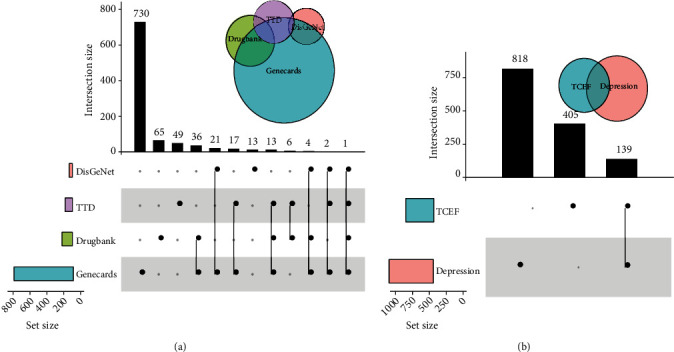
Diseases and common targets of diseases and TCEF. (a) The UpSetR diagram of the disease targets. The horizontal histogram indicates the number of targets included in each disease database. The vertical histogram indicates the number of intersecting targets for the four disease databases represented by the black nodes. (b) The UpSetR diagram of the common targets for TCEF and depression. The horizontal histogram indicates the number of targets included in TCEF and depression. The vertical histogram indicates the number of intersecting targets for the TCEF and depression represented by the black nodes.

Using “depression” as the keyword, we searched four disease target databases (GeneCards, TTD, DisGeNET, and DrugBank) and integrated 1078 depression targets, including 824 targets from GeneCards, 125 from DrugBank, 41 from DisGeNET, and 88 from TTD. The final 957 depression-related targets were obtained by eliminating duplicates (Figure 6(a)). Among them, 139 TCEF common targets with depression were identified for further mechanistic studies (Figure 6(b)).

### 3.6. PPI Network

**Figure 7:**
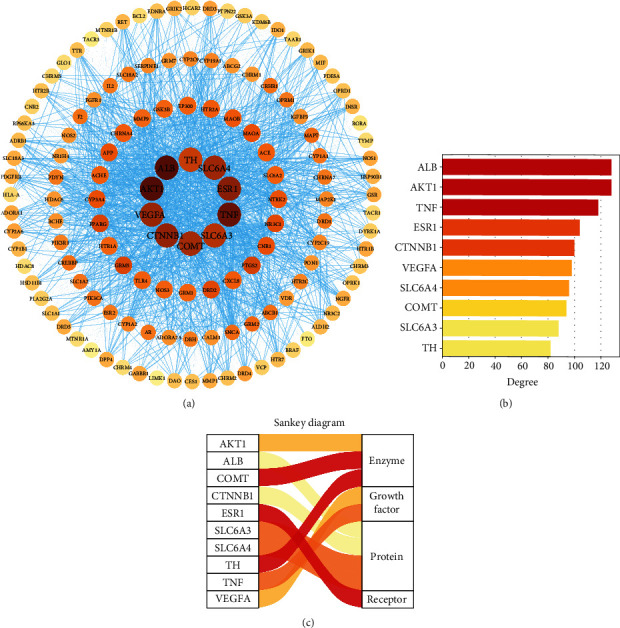
The interrelationship between the targets of TCEF treating depression. (a) PPI network of potential targets. Each node represents a target, and each blue edge indicates the interaction between two nodes. The deeper the color, the greater the degree value. (b) The top 10 core targets were ranked by degree. The horizontal axis is the degree value of the target point, and the vertical axis is the name of the target. The deeper the color, the greater the degree value. (c) Sankey diagram of the top 10 core targets. The color of the Sankey diagram represents its corresponding functional classification.

PPI diagrams were constructed to analyze the interrelationship between the targets (Figure 7(a)). The 139 common targets were uploaded to the String database, the organization was set to “*Homo sapiens*,” 1 free target was deleted, 138 targets were obtained, and the file was saved in the tsv format and uploaded to the Cytoscape 3.8.2 software for network analysis. The network contained 138 nodes and 2730 edges with a mean degree of 39.6, among which ALB, AKT1, TNF, ESR1, CTNNB1, VEGFA, SLC6A4, COMT, SLC6A3, and TH were the top 10 targets in terms of degree (Figure 7(b)). It mainly involves enzymes, proteins, cytokines, and receptors (Figure 7(c)).

### 3.7. GO Functional Enrichment

**Figure 8:**
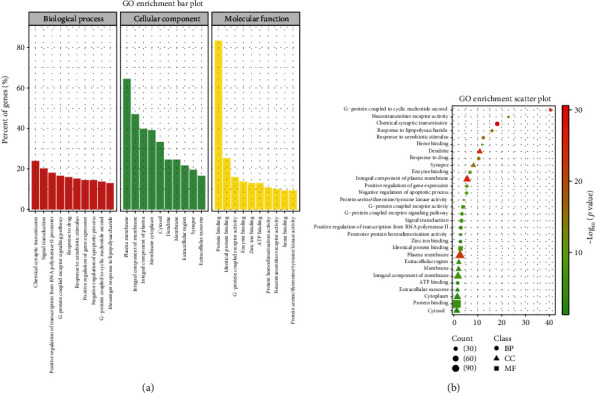
GO analysis of the potential targets of action of TCEF for depression. (a) The top 10 GO enrichment bar plot (BP, CC, and MF). The *X*-axis indicates the name of the GO entries, and the *Y*-axis indicates the count of gene enrichment (%). Different colors indicate the three classifications of GO function, red means BP, green means CC, and yellow means MF. (b) The top 10 GO enrichment scatter plot (BP, CC, and MF). The *X*-axis represents the enrichment factor, the *Y*-axis shows the name of the GO entry. The bubble size represents the count of enrichment targets, and the color represents the *P* value.

The DAVID database was used to enrich the GO classification analysis of 138 potential action targets, and 761 GO entries were identified. Among them, 527 entries were related to biological processes (BP), mainly involving chemical synaptic transmission, signal transduction, positive regulation of transcription from RNA polymerase II promoter, etc. There were 83 entries related to cell composition (CC), mainly relating to the plasma membrane, an integral component of the membrane, and integral component of the plasma membrane. There were 151 entries related to molecular function (MF), mainly relating to protein binding, identical protein binding, and G-protein coupled receptor activity. The top 10 enriched pathways for BP, CC, and MF genes were visualized (Figure 8).

### 3.8. KEGG Enrichment Analysis

**Figure 9:**
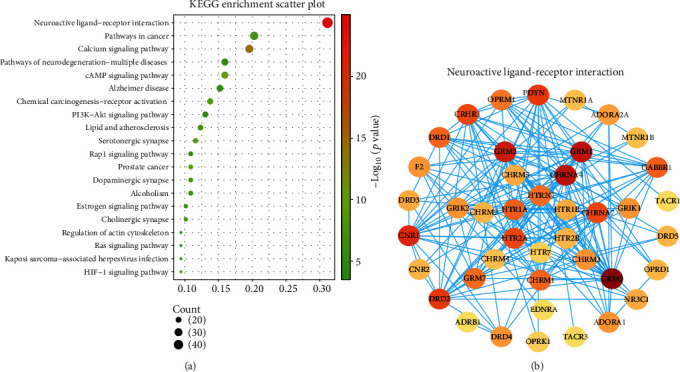
KEGG analysis of the potential targets of action of TCEF for treating depression. (a) The top 20 KEGG enrichment scatter plot. The *X*-axis represents the enrichment factor, the *Y*-axis shows the name of the KEGG entry. The bubble size represents the count of enrichment targets, and the color represents the *P* value. (b) PPI network construction of the neuroactive ligand-receptor interaction. Nodes represent the target; the deeper the color, the greater the role. Edge symbolizes the interaction between two nodes; the thicker the edge, the greater the role.

The DAVID was used to analyze 138 potential targets, and 140 KEGG signaling pathways were obtained. The top 20 pathways were visualized, mainly involving depression-related pathways, such as neuroactive ligand-receptor interaction, calcium signaling pathway, cAMP signaling pathway, PI3K-Akt signaling pathway, serotonergic synapse, and dopaminergic synapse (Figure 9(a)). The neuroactive ligand-receptor interaction is the first pathway enriched by KEGG, and the enriched targets of this pathway mainly involve 5-hydroxytryptamine, dopamine, cholinergic, and glutamate receptors, which are involved in depression-related pathogenesis (Figure 9(b)).

### 3.9. Molecular Docking Verification

**Figure 10:**
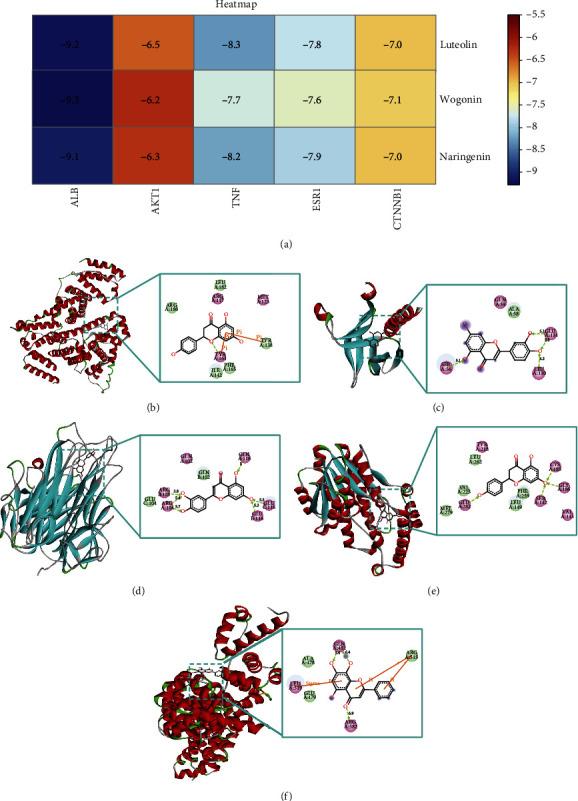
Molecular docking pattern of the target protein-active component of TCEF for depression. (a) Heat map of molecular docking. The bluer the color, the lower the binding energy and the more stable the molecular docking result. (b) Diagram of the interplay between ALB and wogonin. (c) Diagram of the interplay between AKT1 and luteolin. (d) Diagram of the interplay between TNF and luteolin. (e) Diagram of the interplay between ESR1 and naringenin. (f) Diagram of the interplay between CTNNB1 and wogonin. Boxes with dashed lines represent docked active sites; boxes with solid lines represent 2D interactions.

In general, it is believed that the lower the energy at which the ligand binds to the receptor in a stable conformation, the greater the likelihood of an effect occurring. The core compounds, luteolin, wogonin, and naringenin, were molecularly docked with the top five potential targets in terms of degree value, ALB, AKT1, TNF, ESR1, and CTNNB1. The results indicated that the docking binding energy was <−5.0 kJ/mol (Figure 10(a)), suggesting that these active ingredients have a strong binding capacity to their targets [29]. The optimal chimeric structure of the core compound with the core target is presented in Figures 10(b)–10(f). Among them, ALB binds best to wogonin with a binding energy of −9.3 kcal/mol and is linked to ARG-117, TYR-161 in a hydrogen bond, Pi-Pi Stacked with TYR-161, and TYR-138. AKT1 binds best to luteolin with a binding energy of −6.5 kcal/mol and forms hydrogen bonds with GLU-114, LEU-110, and SER-56. TNF binds best to luteolin with a binding energy of −8.3 kcal/mol and is linked to GLU-116, GLU-104, and ARG-103 in the form of hydrogen bonds. ESR1 binds best to naringenin with a binding energy of −7.9 kcal/mol and is linked to GLU-282, SER-142, CYS-185, and GLY-186 in a hydrogen bond. CTNNB1 has the strongest interaction with wogonin, forming hydrogen bonds with GLN-482, ARG-582, PiCation with ARG-515, and PiSigma with LEU-519 at a binding energy of −7.1 kcal/mol.

## 4. Discussion

In this study, the safety of TCEF was first investigated using the maximum dose method. No statistical difference was observed in the visceral adiposity index, body mass, and biochemical blood indices (*P* > 0.05), indicating that TCEF has low oral toxicity and high administration safety. Meanwhile, the pharmacodynamic evaluation of TCEF was performed, and the depression model was successfully established using CUMS. The results of behavioral experiments after drug administration indicated that the TCEF group had a significant decrease in tail suspension immobility time and an increase in sugar water consumption rate compared with the model group, suggesting that TCEF has antidepressant effects.

Chinese herbs are characterized by multiple components, multiple targets, and multiple synergistic pathways, so it is difficult to study their mechanism of action. Therefore, the introduction of network pharmacology can clearly analyze the therapeutic pathways and mechanisms of traditional Chinese herbs by conducting research from a holistic, multilevel, and multilevel perspective [30]. However, most of the traditional network pharmacology studies on the components of traditional Chinese herbs are based on online databases only, and the corresponding targets are mainly obtained by superimposing the predicted targets of every single herbal component, but in the process of actual experimental and clinical use, different herbal concoctions and different decoction methods and times can affect the efficacy of the drugs, so the components of the experimental or clinical use of Chinese herbs are more complex [31–33]. Therefore, in this study, we simulated the common experimental and clinical drug delivery methods to obtain TCEF aqueous decoction, and based on the UHPLC-Q-EOMS technique, 59 chemical components contained in TCEF were analyzed and identified, and 48 compounds were further screened using SwissADME and SwissTargetPrediction for their pharmacological activity. This resulted in more reliable predictions of the exerted pharmacological components than relying exclusively on online databases to obtain the active ingredients [34, 35].

From the TCEF-active ingredient target interaction network analysis, it can be speculated that luteolin, wogonin, and naringenin are the key chemical components of TCEF for treating depression. Luteolin belongs to the group of flavonoids and is an important natural polyphenol found in various plants. The antidepressant activity of luteolin has been reported to be associated with the inhibition of endoplasmic reticulum stress, improvement of oxidative homeostasis, and neuroplasticity [36, 37]. Wogonin competitively inhibits MAO-A and MAO-B [38]. It affects individual sensation, mood, and behavior by regulating the concentration of monoamines, such as 5-hydroxytryptamine, norepinephrine, and dopamine, in the brain and peripheral tissues [39, 40]. Naringenin alleviates depression by regulating oxidative stress and the expression of nuclear factor-*κ*B (NF-*κ*B) and brain-derived neurotrophic factor and by modulating HPA axis dysfunction [41].

Based on the network topology combined with the PPI analysis, we obtained that TCEF can regulate depression through five core targets, namely, ALB, AKT1, TNF, ESR1, and CTNNB1. It was found that the ALB levels were lower in depressed patients than in normal controls, and it is believed that ALB may exert antidepressant effects by regulating oxidative stress responses [42]. Akt is a serine/threonine protein kinase and an important target downstream of PI3K. Akt has three isoforms, AKT1, AKT2, and AKT3, which play an important role in depression. Akt activity is significantly reduced in the brain tissue of depressed patients. Akt enhances hippocampal stem cell function and contributes to synaptic plasticity and neurotransmission formation [43–47]. TNF is involved in depression through several mechanisms, including activation of 5-hydroxytryptamine transporter proteins to mediate the depletion of the active fraction of 5-HT, disruption of the negative feedback loop of the HPA axis by stimulating the excessive release of corticotrophin-releasing hormone, contribution to increased nitric oxide (NO) redox signaling, and participation in hippocampal neurogenesis [48–50]. ESR1 is an estrogen receptor, and estrogen can directly affect brain function through estrogen receptors in the brain regions, which can increase the concentration of various neurotransmitters at the synapses and thus affect their metabolism. Therefore, changes in ESR1 expression may regulate estrogen signaling and further influence the susceptibility of the body to depression. ESR1 has been found to regulate the behavior of depressed mice through the Wnt/b-catenin signaling pathway [51].

GO enrichment analysis revealed that the processes of depression treatment with TCEF mainly involved chemical synaptic transmission, signal transduction, plasma membrane, an integral component of the membrane, and protein binding. This may be related to the complex pathogenesis of depression, suggesting that TCEF can intervene in the development of depression from different levels and multiple pathways.

KEGG enrichment analysis revealed that neuroactive ligand–receptor interaction (hsa04080), cAMP signaling pathway (hsa04024), PI3K-Akt signaling pathway (hsa04151), serotonergic synapse (hsa04726), and dopaminergic synapse (hsa04728) are rich in many targets that may contribute to the treatment of depression with TCEF. The neuroactive ligand–receptor interaction is the pathway with the highest enrichment of targets among the predicted pathways, including 5-hydroxytryptamine receptors, dopamine receptors, cholinergic receptors, adrenergic receptors, and opioid receptors. This pathway is directly related to neurological function and contributes to extracellular signaling into cells, playing a key role in processes such as learning memory capacity, neuroplasticity, and synaptic function, and is associated with the mechanisms of depression and antidepressant effects of many herbal formulations [52–55]. Serotonergic synapse and Dopaminergic synapse pathways are associated with neurotransmitters. The serotonergic synapse pathway consists of 5-hydroxytryptamine and various types of 5-hydroxytryptamine receptors distributed on the presynaptic and postsynaptic membranes in different parts of the brain. Studies have demonstrated that depression is strongly associated with 5-HT receptors and their function [56, 57]. Pleasure deficit disorder is considered a central feature of depression, and dopamine, a monoamine transmitter, is an important neurotransmitter in the hypothalamus and pituitary gland, and the dopaminergic synapse pathway plays a key role in the euphoria deficit described by this depression [58].

Calcium, cAMP, and PI3K-Akt signaling pathways are associated with signaling. Depression has been found to be associated with the dysregulation of divalent calcium ions, which act mainly through the calcium signaling cascade. The calcium signaling pathway stimulates NO synthesis, which then induces guanylate cyclase to convert guanosine triphosphate to cGMP. Calcium imbalance leads to neuronal apoptosis, which then alters the structure and function of the hippocampal region, ultimately leading to depression [59]. cAMP signaling enhances the excitability of neural circuits and has a facilitative effect on memory. Activation of the cAMP pathway enhances hippocampal neuron regeneration in mice with a depressive-like behavior [60]. The PI3K/Akt signaling pathway is the main signaling pathway through which central neuroprotective agents act, and it is one of the more maturely studied pathways. Studies have demonstrated that PI3K/Akt is associated with depression, and regulating this signaling pathway can exert antidepressant effects by regulating brain-derived neurotrophic factors and their receptors, regulating glutamate receptors and their transporter proteins, and inhibiting neuronal apoptosis [61, 62].

The molecular docking results indicated that the effective core compounds of TCEF had binding energies <−5 kJ/mol to the core target proteins ALB, AKT1, TNF, ESR1, and CTNNB1, and there were multiple binding sites with the amino acid residues of the core targets, forming hydrogen-bonding interactions. The high binding activities of these core compounds to the receptor proteins suggest that the results of this study are relatively accurate and reliable and can provide a theoretical basis for the treatment of depression with TECF at the molecular level. At the same time, there are some shortcomings in this study. The core targets and pathways predicted by the network pharmacology are not supported by relevant cellular and animal experimental data, and follow-up studies will experimentally validate the key targets and pathways through which TCEF plays an antidepressant role.

## 5. Conclusion

We have experimentally demonstrated that TCEF has a safety profile and antidepressant effects. Furthermore, we preliminarily identified the chemical composition of TCEF using UHPLC-Q-EOMS. Using the method of network pharmacology and molecular docking, the mechanism of TCEF antidepression through multicomponent, multitarget, and multipathway was predicted. TCEF mainly regulates neuroactive ligand-receptor interaction, calcium signaling pathway, cAMP signaling pathway, PI3K-Akt signaling pathway, serotonergic synapse, and dopaminergic synapse pathway, acting on core targets, such as ALB, AKT1, TNF, ESR1, and CTNNB1, to regulate neurotransmitters and their receptors, enhance neurotrophy and protection, and reduce neuronal apoptosis damage, exerting antidepressant effects.

## Figures and Tables

**Table 1:** UHPLC-Q-EOMS of TCEF chemical composition identification.

No.	Name	Formula	Molecular weight	RT [min]	Mass error (*δ*)/ppm	Mode	Major fragment ions
1	DL-arginine	C_6_H_14_N_4_O_2_	174.1117	0.758	-0.06	[M + H]^+^	70.06572,157.10812
2	Choline	C_5_H_13_NO	103.1	0.799	2.78	[M + H]^+^	58.06576,104.10726
3	DL-stachydrine	C_7_H_13_NO_2_	143.0946	0.821	-0.58	[M + H]^+^	84.08128,144.10188
4	Pipecolic acid	C_6_H_11_NO_2_	129.079	0.839	0.26	[M + H]^+^	84.08125, 130.08626
5	3,4,5-Trihydroxycyclohex-1-ene-1-carboxylic acid	C_7_H_10_O_5_	174.0521	0.878	-4.03	[M-H]^−^	93.03362,111.04424,143.03448
6	Pyrogallol	C_6_H_6_O_3_	126.0318	0.976	0.67	[M + H]^+^	71.04972, 109.02869
7	Citric acid	C_6_H_8_O_7_	192.0265	1.039	-2.66	[M-H]-	111.00784,191.01923
8	Nicotinic acid	C_6_H_5_NO_2_	123.0322	1.044	1.05	[M + H]^+^	78.03440,124.03943
9	Nicotinamide	C_6_H_6_N_2_O	122.0482	1.045	1.38	[M + H]^+^	106.02934,123.05547
10	Adenosine	C_10_H_13_N_5_O_4_	267.0956	1.046	-4.44	[M + H]^+^	115.03914,136.06172
11	L-tyrosine	C_9_H_11_NO_3_	181.0739	1.048	0.05	[M + H]^+^	136.07576,182.08124
12	Acetophenone	C_8_H_8_O	120.0577	1.078	1.34	[M + H]^+^	77.03935,103.05459
13	DL-norleucine	C_6_H_13_NO_2_	131.0947	1.126	0.64	[M + H]^+^	73.06535,132.10188
14	4-Pyridoxic acid	C_8_H_9_NO_4_	183.0527	1.178	-2.32	[M-H]^−^	94.02896,138.05519
15	3-Hydroxy-3-methylglutaric acid	C_6_H_10_O_5_	162.0522	1.181	-3.67	[M-H]^−^	57.03351,99.04419
16	Gallic acid	C_7_H_6_O_5_	170.021	1.284	-3.08	[M-H]^−^	67.01773,125.02354
17	Tetramethylpyrazine	C_8_H_12_N_2_	136.1002	1.493	0.94	[M + H]^+^	137.10732,122.08398
18	3-Hydroxymandelic acid	C_8_H_8_O_4_	168.0418	1.905	-2.96	[M-H]^−^	109.02872,123.04429
19	Pantothenic acid	C_9_H_17_NO_5_	219.1104	1.94	-1.32	[M + H]^+^	116.03443, 202.10736, 222.11737
20	2,3-Dihydroxybenzoic acid	C_7_H_6_O_4_	154.026	2.081	-3.86	[M-H]^−^	65.03860,109.02859
21	2,4-Dihydroxybenzoic acid	C_7_H_6_O_4_	154.026	2.2	-3.71	[M-H]^−^	65.03860,109.02856
22	2-Methoxyresorcinol	C_7_H_8_O_3_	140.0474	2.306	0.15	[M + H]^+^	67.05480,127.03905
23	Sorbic acid	C_6_H_8_O_2_	112.0528	2.731	2.91	[M + H]^+^	57.07050,113.05990
24	Gentisic acid	C_7_H_6_O_4_	154.026	3.348	-3.86	[M-H]^−^	109.02857,153.01846
25	2-Isopropylmalic acid	C_7_H_12_O_5_	176.0679	3.57	-3.26	[M-H]^−^	85.06489,115.03917,141.05457
26	Mescaline	C_11_H_17_NO_3_	211.1207	3.69	-0.9	[M + H]^+^	103.05458,195.10155
27	Catechin	C_15_H_14_O_6_	290.0791	3.709	0.08	[M-H]^−^	205.05017,245.08185
28	3,4-Dihydroxybenzaldehyde	C_7_H_6_O_3_	138.0315	3.712	-1.44	[M + H]^+^	83.04970,93.03407
29	BMK methyl glycidate	C_11_H_12_O_3_	192.0788	3.878	0.56	[M + H]^+^	79.05482,161.05971
30	N-acetyltyramine	C_10_H_13_NO_2_	179.0946	3.888	-0.04	[M + H]^+^	121.06493,180.10187
31	4-Methoxysalicylic acid	C_8_H_8_O_4_	168.0424	4.047	0.79	[M + H]^+^	111.00792,169.04953
32	4-Hydroxybenzaldehyde	C_7_H_6_O_2_	122.0372	4.576	3.41	[M + H]^+^	53.03933,95.04959
33	Syringic acid	C_9_H_10_O_5_	198.0529	4.641	0.41	[M + H]^+^	125.02370,140.04686,
34	N-acetyl-D-alloisoleucine	C_8_H_15_NO_3_	173.1048	5.396	-2.47	[M + H]^+^	130.08648,172.09727
35	Vanillin	C_8_H_8_O_3_	152.0474	5.725	0.34	[M + H]^+^	93.03390,125.05982,153.05475
36	Esculetin	C_9_H_6_O_4_	178.0266	6.172	-0.15	[M + H]^+^	117.03374,179.03381
37	DL-mandelic acid	C_8_H_8_O_3_	152.0466	6.177	-4.7	[M + H]^+^	136.01628,151.03928
38	N-acetyl-L-phenylalanine	C_11_H_13_NO_3_	207.0893	6.522	-1.36	[M + H]^+^	91.05433,104.07098
39	Suberic acid	C_8_H_14_O_4_	174.0887	6.949	-3.11	[M + H]^+^	69.03326,129.09102,178.08131
40	Ferulic acid	C_10_H_10_O_4_	194.058	7.12	0.3	[M + H]^+^	89.03897,145.02840
41	Senkyunolide H	C_12_H_16_O_4_	224.1047	7.325	-0.57	[M + H]^+^	133.10121, 161.09604, 189.09102
42	Resveratrol	C_14_H_12_O_3_	228.0783	7.426	-1.73	[M + H]^+^	95.04979, 135.04407, 229.08528
43	Naringenin	C_15_H_12_O_5_	272.0676	7.629	-3.39	[M + H]^+^	123.04433,273.07480
44	N-acetyl-DL-tryptophan	C_13_H_14_N_2_O_3_	246.1005	7.701	0.08	[M + H]^+^	74.02369,203.08228
45	Dehydroacetic acid	C_8_H_8_O_4_	168.0414	7.931	-4.87	[M + H]^+^	123.04424,167.03426
46	4-Indolecarbaldehyde	C_9_H_7_NO	145.0524	8.127	-2.72	[M + H]^+^	91.05465,118.06533
47	3′,4′-Dihydroxyphenylacetone	C_9_H_10_O_3_	166.0629	9.541	-0.79	[M + H]^+^	91.05453,149.05969,167.07022
48	Phloretin	C_15_H_14_O_5_	274.083	9.826	-4.12	[M + H]^+^	107.04948, 275.08960
49	Cynaroside	C_21_H_20_O_11_	448.1008	11.754	0.43	[M + H]^+^	285.04056,447.09290
50	(±)-Abscisic acid	C_15_H_20_O_4_	264.1353	12.092	-3.12	[M + H]^+^	229.12219
51	3-tert-Butyladipic acid	C_10_H_18_O_4_	202.1202	12.635	-1.57	[M + H]^+^	57.03331,139.11200
52	(5S,6S)-5-Hydroxy-4-methoxy-6-[(E)-2-phenylvinyl]-5,6-dihydro-2H-pyran-2-one	C_14_H_14_O_4_	246.0884	13.465	-3.38	[M + H]^+^	229.08531,
53	Ambrosic acid	C_15_H_20_O_4_	264.1361	15.962	-0.14	[M + H]^+^	201.12773,219.13884
54	Emodin	C_15_H_10_O_5_	270.0516	16.58	-4.45	[M + H]^+^	95.08553
55	Wogonin	C_16_H_12_O_5_	284.0672	19.668	-4.53	[M + H]^+^	270.05112,285.07434
56	Genistein	C_15_H_10_O_5_	270.0527	22.102	-0.63	[M + H]^+^	225.05544,269.04556
57	Luteolin	C_15_H_10_O_6_	286.0478	23.603	0.34	[M + H]^+^	285.0405
58	Stearamide	C_18_H_37_NO	283.2863	33.9	-4.17	[M + H]^+^	284.29340,95.08595
59	Maltol	C_6_H_6_O_3_	126.0318	38.768	0.67	[M + H]^+^	109.02869,127.03906

## Data Availability

The datasets presented in this study are accessible in the article/Supplement Material.

## Supplementary Materials

Supplementary Materials Supplementary Table 1: composition of TCEF screened by SwissADME. Table 2: composition of TCEF screened by SwissTargetPrediction.
