# Supplementary material for: Exploring the Mechanism of Action of Trachelospermi Caulis et Folium for Depression Based on Experiments: Combining Network Pharmacology and Molecular Docking
Source: Comput Math Methods Med. 2022 Nov 30;2022:3945063. doi: 10.1155/2022/3945063 (PMC9729047; doi:10.1155/2022/3945063)
Supplement: Supplementary Materials — Supplementary Table 1: composition of TCEF screened by SwissADME. Table 2: composition of TCEF screened by SwissTargetPrediction. [file 3945063.f1.docx]

Supplementary Material

# Supplementary Tables

Supplementary Table 1. Composition of TCEF screened by SwissADME.

| **No.** | **Name** | **Formula** |
| --- | --- | --- |
| 1 | Wogonin | C_16_H_12_O_5_ |
| 2 | Vanillin | C_8_H_8_O_3_ |
| 3 | Tetramethylpyrazine | C_8_H_12_N_2_ |
| 4 | Syringic acid | C_9_H_10_O_5_ |
| 5 | Suberic acid | C_8_H_14_O_4_ |
| 6 | Sorbic acid | C_6_H_8_O_2_ |
| 7 | Senkyunolide H | C_12_H_16_O_4_ |
| 8 | Resveratrol | C_14_H_12_O_3_ |
| 9 | Pyrogallol | C_6_H_6_O_3_ |
| 10 | Pipecolic acid | C_6_H_11_NO_2_ |
| 11 | Phloretin | C_15_H_14_O_5_ |
| 12 | Pantothenic acid | C_9_H_17_NO_5_ |
| 13 | Nicotinic acid | C_6_H_5_NO_2_ |
| 14 | Nicotinamide | C_6_H_6_N_2_O |
| 15 | Naringenin | C_15_H_12_O_5_ |
| 16 | N-Acetyltyramine | C_10_H_13_NO_2_ |
| 17 | N-Acetyl-L-phenylalanine | C_11_H_13_NO_3_ |
| 18 | N-Acetyl-DL-tryptophan | C_13_H_14_N_2_O_3_ |
| 19 | N-Acetyl-D-alloisoleucine | C_8_H_15_NO_3_ |
| 20 | Mescaline | C_11_H_17_NO_3_ |
| 21 | Maltol | C_6_H_6_O_3_ |
| 22 | Luteolin | C_15_H_10_O_6_ |
| 23 | L-Tyrosine | C_9_H_11_NO_3_ |
| 24 | Gentisic acid | C_7_H_6_O_4_ |
| 25 | Genistein | C_15_H_10_O_5_ |
| 26 | Gallic acid | C_7_H_6_O_5_ |
| 27 | Ferulic acid | C_10_H_10_O_4_ |
| 28 | Esculetin | C_9_H_6_O_4_ |
| 29 | Emodin | C_15_H_10_O_5_ |
| 30 | DL-Stachydrine | C_7_H_13_NO_2_ |
| 31 | DL-Norleucine | C_6_H_13_NO_2_ |
| 32 | DL-Mandelic acid | C_8_H_8_O_3_ |
| 33 | Dehydroacetic acid | C_8_H_8_O_4_ |
| 34 | Catechin | C_15_H_14_O_6_ |
| 35 | BMK methyl glycidate | C_11_H_12_O_3_ |
| 36 | Ambrosic acid | C_15_H_20_O_4_ |
| 37 | Acetophenone | C_8_H_8_O |
| 38 | 4-Pyridoxic acid | C_8_H_9_NO_4_ |
| 39 | 4-Methoxysalicylic acid | C_8_H_8_O_4_ |
| 40 | 4-Indolecarbaldehyde | C_9_H_7_NO |
| 41 | 4-Hydroxybenzaldehyde | C_7_H_6_O_2_ |
| 42 | 3-tert-Butyladipic acid | C_10_H_18_O_4_ |
| 43 | 3-Hydroxymandelic acid | C_8_H_8_O_4_ |
| 44 | 3-Hydroxy-3-methylglutaric acid | C_6_H_10_O_5_ |
| 45 | 3',4'-Dihydroxyphenylacetone | C_9_H_10_O_3_ |
| 46 | 3,4-Dihydroxybenzaldehyde | C_7_H_6_O_3_ |
| 47 | 3,4,5-trihydroxycyclohex-1-ene-1-carboxylic acid | C_7_H_10_O_5_ |
| 48 | 2-Methoxyresorcinol | C_7_H_8_O_3_ |
| 49 | 2-Isopropylmalic acid | C_7_H_12_O_5_ |
| 50 | 2,4-Dihydroxybenzoic acid | C_7_H_6_O_4_ |
| 51 | 2,3-Dihydroxybenzoic acid | C_7_H_6_O_4_ |
| 52 | (5S,6S)-5-Hydroxy-4-methoxy-6-[(E)-2-phenylvinyl]-5,6-dihydro-2H-pyran-2-one | C_14_H_14_O_4_ |
| 53 | (±)-Abscisic acid | C_15_H_20_O_4_ |

Supplementary Table 2. Composition of TCEF screened by SwissTargetPrediction.

| **TCEF No.** | **Name** | **Formula** |
| --- | --- | --- |
| TCEF1 | Wogonin | C_16_H_12_O_5_ |
| TCEF2 | Vanillin | C_8_H_8_O_3_ |
| TCEF3 | Syringic acid | C_9_H_10_O_5_ |
| TCEF4 | Suberic acid | C_8_H_14_O_4_ |
| TCEF5 | Sorbic acid | C_6_H_8_O_2_ |
| TCEF6 | Resveratrol | C_14_H_12_O_3_ |
| TCEF7 | Pyrogallol | C_6_H_6_O_3_ |
| TCEF8 | Pipecolic acid | C_6_H_11_NO_2_ |
| TCEF9 | Phloretin | C_15_H_14_O_5_ |
| TCEF10 | Pantothenic acid | C_9_H_17_NO_5_ |
| TCEF11 | Nicotinic acid | C_6_H_5_NO_2_ |
| TCEF12 | Nicotinamide | C_6_H_6_N_2_O |
| TCEF13 | Naringenin | C_15_H_12_O_5_ |
| TCEF14 | N-Acetyltyramine | C_10_H_13_NO_2_ |
| TCEF15 | N-Acetyl-L-phenylalanine | C_11_H_13_NO_3_ |
| TCEF16 | N-Acetyl-DL-tryptophan | C_13_H_14_N_2_O_3_ |
| TCEF17 | N-Acetyl-D-alloisoleucine | C_8_H_15_NO_3_ |
| TCEF18 | Mescaline | C_11_H_17_NO_3_ |
| TCEF19 | Maltol | C_6_H_6_O_3_ |
| TCEF20 | Luteolin | C_15_H_10_O_6_ |
| TCEF21 | L-Tyrosine | C_9_H_11_NO_3_ |
| TCEF22 | Gentisic acid | C_7_H_6_O_4_ |
| TCEF23 | Genistein | C_15_H_10_O_5_ |
| TCEF24 | Gallic acid | C_7_H_6_O_5_ |
| TCEF25 | Ferulic acid | C_10_H_10_O_4_ |
| TCEF26 | Esculetin | C_9_H_6_O_4_ |
| TCEF27 | Emodin | C_15_H_10_O_5_ |
| TCEF28 | DL-Stachydrine | C_7_H_13_NO_2_ |
| TCEF29 | DL-Norleucine | C_6_H_13_NO_2_ |
| TCEF30 | DL-Mandelic acid | C_8_H_8_O_3_ |
| TCEF31 | Dehydroacetic acid | C_8_H_8_O_4_ |
| TCEF32 | BMK methyl glycidate | C_11_H_12_O_3_ |
| TCEF33 | Ambrosic acid | C_15_H_20_O_4_ |
| TCEF34 | Acetophenone | C_8_H_8_O |
| TCEF35 | 4-Pyridoxic acid | C_8_H_9_NO_4_ |
| TCEF36 | 4-Methoxysalicylic acid | C_8_H_8_O_4_ |
| TCEF37 | 4-Indolecarbaldehyde | C_9_H_7_NO |
| TCEF38 | 4-Hydroxybenzaldehyde | C_7_H_6_O_2_ |
| TCEF39 | 3-tert-Butyladipic acid | C_10_H_18_O_4_ |
| TCEF40 | 3-Hydroxymandelic acid | C_8_H_8_O_4_ |
| TCEF41 | 3-Hydroxy-3-methylglutaric acid | C_6_H_10_O_5_ |
| TCEF42 | 3',4'-Dihydroxyphenylacetone | C_9_H_10_O_3_ |
| TCEF43 | 3,4-Dihydroxybenzaldehyde | C_7_H_6_O_3_ |
| TCEF44 | 3,4,5-trihydroxycyclohex-1-ene-1-carboxylic acid | C_7_H_10_O_5_ |
| TCEF45 | 2-Methoxyresorcinol | C_7_H_8_O_3_ |
| TCEF46 | 2-Isopropylmalic acid | C_7_H_12_O_5_ |
| TCEF47 | (±)-Abscisic acid | C_7_H_6_O_4_ |
| TCEF48 | 2,4-Dihydroxybenzoic acid | C_15_H_20_O_4_ |
